# Room-Temperature Aqueous Synthesis of Copper Nanoparticles and Their In Situ Conversion to Copper Azides

**DOI:** 10.3390/mi17070763

**Published:** 2026-06-23

**Authors:** Chang Leng, Mingyu Li, Qingxuan Zeng, Pengfei Xue, Jie Ren, Zhenhao Shi, Yu Zhou, Zhongcai Li

**Affiliations:** 1 State Key Laboratory of Explosion Science and Safety Protection, School of Mechatronical Engineering, Beijing Institute of Technology, Beijing 100081, China; 2 China Research and Development Academy of Machinery Equipment, Beijing 100089, China

**Keywords:** copper nanoparticles, copper azides, in situ gas–solid reaction, NTA

## Abstract

Copper azides are promising energetic materials for miniaturized pyrotechnic devices and micro explosive trains owing to their short detonation growth distance and high initiation energy. However, controllable preparation of copper nanoparticle precursors and their in situ conversion to copper azides under mild conditions remains challenging. In this study, copper nanoparticles were synthesized via a coordination-assisted aqueous reduction method at room temperature under air atmosphere using nitrilotriacetic acid disodium salt (NTA·H·2Na) as the complexing agent. The resulting nanoparticles were pressed into polyester rings to construct confined precursor structures, and copper azide micro-charges were prepared through in situ gas–solid reaction with HN_3_ gas generated from NaN_3_ and concentrated phosphoric acid at 60 °C. SEM characterization revealed that the morphological evolution of copper azides followed a three-stage pattern: “product island nucleation, branch/block coalescence growth, and continuous product layer formation and structural reconstruction”. Detonation velocity tests using the electrical probe method showed an average value of (5.10 ± 0.07) × 10^3^ m/s. Flyer impact initiation tests demonstrated that, with a charge thickness of 1.00 mm, both a 30 μm polyimide flyer and a 40 μm titanium flyer could successfully initiate a HNS–IV explosive. The preparation methodology and performance characterization established in this work provide an experimental basis for the application of copper azides in micro-initiation systems.

## 1. Introduction

Copper azides, as energetic materials with high energy density, have significant application potential in miniaturized pyrotechnic devices and micro explosive trains owing to their low initiation threshold, small critical dimension, and short detonation growth distance [1,2,3]. Compared with traditional lead-based primary explosives, copper azides are lead-free, environmentally friendly, and possess stronger initiation capability, making them promising candidates as green primary explosives for miniaturized detonation systems [2]. However, their high sensitivity poses considerable safety risks during synthesis, loading, and assembly, which restricts their practical application and engineering development [1,2].

Among various preparation strategies, in situ reaction routes based on copper precursors and hydrazoic acid enable direct generation of copper azides within confined spaces, thereby reducing the exposure risk of highly sensitive energetic products during transfer, loading, and post-processing. This approach has demonstrated strong application potential in the fabrication of micro-initiation components [1,2,4,5,6,7,8,9]. The particle size distribution, agglomeration state, pore structure, and surface chemical characteristics of the precursor significantly influence the diffusion and transport of reactive gases within the particle packing system, as well as the interfacial reaction behavior and ultimate microstructure and initiation performance of the final product [5,6,10,11,12]. Therefore, constructing copper nanoparticle precursors with relatively uniform particle size, good dispersibility, and tunable surface activity is a crucial foundation for achieving controllable in situ gas–solid synthesis of copper azides.

Currently, various methods exist for preparing copper nanoparticles; however, in traditional routes, copper nanoparticles are prone to oxidation, and polymers or organic solvents are often introduced to control particle size, which increases the complexity of practical applications [13,14]. How to simultaneously achieve controlled particle size, good dispersibility, and sufficient stability under mild conditions remains a problem worthy of further investigation. Kamikoriyama et al. reported a method for synthesizing copper nanoparticles in an aqueous phase at room temperature under air atmosphere using a NTA·H·2Na/Cu(OH)_2_ coordination system with hydrazine hydrate as the reductant. The average particle size of the obtained copper nanoparticles was controlled at approximately 50–60 nm, and a Cu_2_O shell/NTA coating structure formed on the particle surface, contributing to improved oxidation resistance [15]. This indicates the feasibility of such routes for precursor construction.

Existing studies have shown that copper azides undergo three stages during the gas–solid azidation process, “product island formation—product island growth and diffusion—Ostwald ripening”, with their reaction pathways exhibiting different modes depending on conditions such as HN_3_ concentration [8]. However, for copper nanoparticle precursors that require press-loading in micro-charge configurations, research on the morphological evolution during in situ gas–solid reaction and its correlation with detonation output performance remains relatively limited. In the design of micro explosive trains, whether the detonation output of copper azides can effectively initiate the subsequent booster explosive is an important criterion for evaluating their engineering application value. HNS–IV explosive, owing to its high thermal stability and low mechanical sensitivity, is commonly used as a booster explosive to evaluate the output performance of micro-initiation devices [16,17].

Based on the above understanding, this paper employs an aqueous-phase reduction method at room temperature under air atmosphere to prepare copper nanoparticles, introducing the multidentate complexing agent NTA·H·2Na to regulate the coordination environment and reactivity of the Cu(II) precursor, with the aim of obtaining copper nanoparticle samples with narrow particle size distribution and low agglomeration. On this basis, copper nanoparticles are pressed into polyester rings to construct precursor structures, and copper azides are synthesized through in situ gas–solid reaction with HN_3_ gas. The precursor synthesis process conditions, gas–solid reaction morphological evolution, detonation output performance, and impact of initiation capability on HNS–IV explosives are systematically investigated, providing an experimental basis for the engineering application of copper azide micro-charges in micro-initiation trains.

## 2. Materials and Methods

### 2.1. Materials and Instruments

The main chemical reagents and instruments used in this study are listed in Table 1 and Table 2, respectively.

### 2.2. Synthesis of Copper Nanoparticles

Copper nanoparticles were synthesized in an aqueous system at room temperature under air atmosphere. A water-soluble Cu(II)–NTA complex precursor was formed by reacting NTA·H·2Na with Cu(OH)_2_, which was then reduced by hydrazine hydrate to generate copper nanoparticles. NTA, as a multidentate complexing agent, can regulate the reactivity of Cu(II) through complexation, thereby influencing the nucleation and growth behavior during the reduction process and facilitating the formation of copper nanoparticles with a narrow particle size distribution.

The specific steps were as follows: In a 1 L round-bottom flask, 43 mL of NaOH aqueous solution (2.8 mol/L) and 340 mL of NTA·H·2Na aqueous solution (0.71 mol/L) were sequentially added and mixed under magnetic stirring at room temperature (27 ± 2 °C) under air conditions. Subsequently, 11.8 g of Cu(OH)_2_ powder (0.12 mol) was added, and stirring was continued until completely dissolved, forming a dark blue transparent solution. At this point, the molar ratio of NTA·H·2Na to Cu(OH)_2_ was 2.0. Under the same conditions, 98 mL of hydrazine hydrate solution (4.9 mol/L) was added rapidly in one portion, and the stirring speed was adjusted to 300 r/min. The solution quickly changed from dark blue to dark purple, indicating the reduction in Cu(II) and the formation of copper nanoparticles. Stirring was continued for 1 h to ensure complete reduction. After the reaction, the resulting mixture was centrifuged at 10,000 g for 30 min to separate the solid product. The obtained precipitate was washed twice by dispersion in distilled water followed by centrifugation, and washed three times by dispersion in anhydrous ethanol followed by centrifugation, to remove residual ligands and by-products. Finally, the resulting slurry was transferred to a rotary evaporator to remove ethanol at 40 °C and 175 mbar, yielding dry copper nanoparticle powder, which was sealed and stored under vacuum.

For comparison, a control sample was prepared by replacing the NTA·H·2Na solution with an equal amount of distilled water, following the same procedure. This method uses water as the reaction medium under mild operating conditions; however, the reductant hydrazine hydrate is toxic and must be handled in a fume hood with appropriate personal protective equipment.

### 2.3. Characterization Methods

Field Emission Scanning Electron Microscopy (SEM) was used to observe the micromorphology of the copper nanoparticles and copper azides. Energy Dispersive Spectroscopy (EDS) attached to the SEM was used to analyze the elemental composition and distribution in micro-areas of the samples. A nanoparticle size and zeta potential analyzer was used to measure the hydrodynamic size distribution of oxidized copper nanoparticles (exposed to air for one week) dispersed in anhydrous ethanol, assessing their size characteristics in the dispersion system.

### 2.4. In Situ Synthesis of Copper Azides

Preparation of Precursor for Gas–Solid Reaction: The polyester ring was a planar annular structure with a central through-hole, having a thickness of 0.50 mm, an outer diameter of 3.00 mm, and a central through-hole diameter of 1.00 mm. Copper nanoparticles were loaded into the ring by external pressing using a powder tablet press at 0.50 MPa for 2 min, ensuring uniform filling and formation of a structure with a certain strength in the through-hole. Based on the sample mass and geometric dimensions, the actual loading density of copper nanoparticles in the polyester ring in this experiment was 1.50 g/cm^3^, which met the requirements for subsequent initiation performance tests. After loading, the ring was required to meet the following quality criteria: flat upper and lower surfaces without warping or collapse; clean polyester surfaces free of copper powder adhesion or contamination; and the copper nanoparticles in the central through-hole compacted into an integral block structure without obvious voids, delamination, or loose powder.

In Situ Gas–Solid Synthesis Method: HN_3_ gas was generated by reacting NaN_3_ with concentrated phosphoric acid and introduced into the reaction chamber to contact the copper nanoparticles, achieving in situ gas–solid synthesis of copper azides. The generation of HN_3_ can be described by the following reaction equation:

(1) $$3\mathrm{Na}N_{3}\left(s\right)\;+\;H_{3}{\mathrm{PO}}_{4}\left(l\right)\;\overset{60\;{}^{\circ}C}{\to }\;3HN_{3}\left(g\right)\;+\;{\mathrm{Na}}_{3}{\mathrm{PO}}_{4}\left(s\right)$$

The azidation process between copper nanoparticles and HN_3_ gas is a typical non-catalytic gas–solid reaction system. Since the boiling point of HN_3_ is approximately 36 °C, a relatively high concentration of HN_3_ gas can be continuously generated at 60 °C, creating a gas–solid reaction environment with low temperature and high HN_3_ partial pressure. Under low-temperature, high HN_3_ concentration conditions, the reaction between copper and HN_3_ gas typically preferentially forms Cu(N_3_)_2_. The possible reaction process is [8]:

(2) $$\mathrm{Cu}\left(s\right)\;+\;3HN_{3}\left(g\right)\;\to \mathrm{Cu}{\left(N_{3}\right)}_{2}\left(s\right)\;+NH_{3}\left(g\right)\;+\;N_{2}\left(g\right)$$

As the reaction proceeds further, the generated Cu(N_3_)_2_ can undergo a conversion reaction with unreacted metallic copper via solid-phase diffusion and interfacial reactions to form CuN_3_:

(3) $$\mathrm{Cu}\left(s\right)\;+\;\mathrm{Cu}{\left(N_{3}\right)}_{2}\left(s\right)\;\to 2\mathrm{Cu}N_{3}\left(s\right)$$

**Figure 1:**
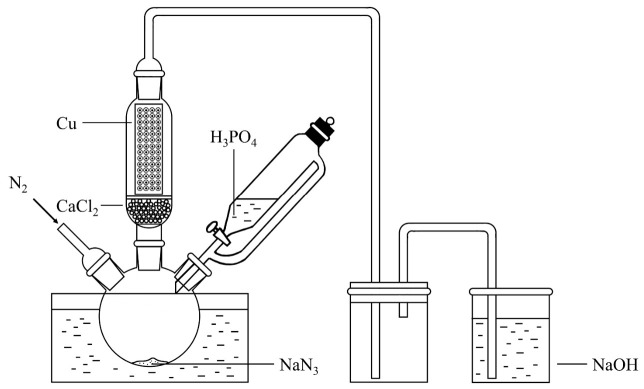
Schematic diagram of the gas–solid in situ reaction apparatus.

A schematic diagram of the gas–solid reaction apparatus is shown in Figure 1. The apparatus mainly includes a gas generator, a dryer, a reaction chamber, a trap bottle, and an exhaust gas absorption system. The gas generator contains solid NaN_3_ and concentrated phosphoric acid; the dryer is filled with anhydrous CaCl_2_ to remove moisture from the HN_3_ gas; the reaction chamber is used to place the polyester ring loaded with copper nanoparticles; the exhaust gas absorption bottle contains NaOH solution to absorb unreacted HN_3_ gas. Components are connected via polytetrafluoroethylene tubing, and the entire apparatus is operated inside a fume hood.

The experimental procedure was as follows: Before the reaction, the airtightness of the apparatus was checked to ensure good sealing and prevent HN_3_ leakage. The polyester ring loaded with copper nanoparticles was fixed onto the reaction plate and then placed inside the reaction chamber. Subsequently, 3 g of NaN_3_ was added to the gas generator, and 10 mL of concentrated phosphoric acid was added to the constant pressure dropping funnel. The water bath heating was turned on to reach the set temperature (60 °C in this experiment), and concentrated phosphoric acid was added dropwise to the NaN_3_ powder at a rate of one drop every 5 s, allowing continuous generation of HN_3_ gas which passed into the reaction chamber to react with the copper nanoparticles. After 12 h of reaction, the water bath heating was turned off, allowing the reaction apparatus to cool to room temperature. After 24 h of total reaction time, the reaction was terminated. N_2_ gas was purged into the apparatus to completely sweep the residual gas into the exhaust gas absorption system. After confirming no HN_3_ residue remained, the reaction plate was removed from the reaction chamber, yielding the copper azide sample confined within the polyester ring.

### 2.5. Performance Test Methods

#### 2.5.1. Detonation Velocity Test

Given the small characteristic size of the copper azide samples and the difficulty in arranging conventional test methods, the electrical probe method was employed to test their detonation velocity. Flexible electrical probes with a metal film coated on one side of a polyimide film (thickness approximately 20 μm) were used. During the test, the flexible electrical probes were placed at preset intervals along the charge propagation path, with the metal film side facing the explosive within the preset interval to avoid errors introduced by film thickness. When the copper azides detonate, the ionization effect of the detonation products conducts the probe circuit, triggering a corresponding conduction signal recorded on the oscilloscope. By measuring the distance between adjacent probes and the time difference in conduction, the average propagation velocity of the detonation wave within that interval can be calculated. The calculation principle uses the ratio of the preset distance Δ*L* to the corresponding conduction time interval Δ*t* as the average detonation velocity for that section:

(4) $$\bar{V}=\Delta L/\Delta t$$

**Figure 2:**
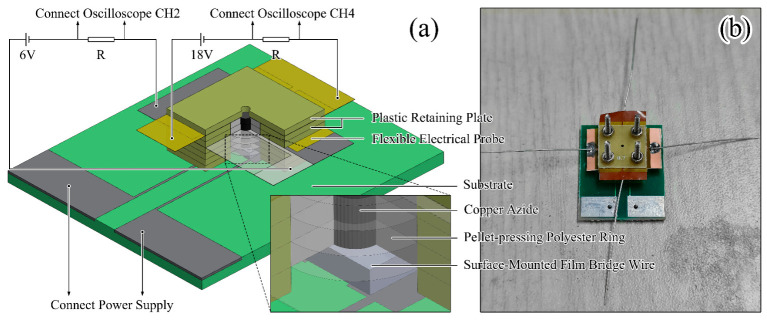
Schematic diagram of the detonation velocity test system. (**a**) Schematic; (**b**) Photograph.

A schematic diagram of the detonation velocity test system using the electrical probe method is shown in Figure 2. The system consists of a test device, an initiation power supply, and two conduction circuits. The test device includes a substrate, a surface-mounted film bridge wire, copper azide samples (5 in total), plastic retaining plates with a central through-hole diameter of 3.00 mm (6 in total), a plastic retaining plate with a central through-hole diameter of 0.70 mm, and flexible electrical probes (2 in total). Each copper azide sample comprises copper azides and the supporting polyester ring. The thickness of a single sample measured by a micrometer screw was 0.50 mm, and the outer diameter was 3.00 mm. The flexible electrical probes have a central through-hole diameter of 0.80 mm and are positioned behind the 3rd and 5th copper azide samples, respectively, with the coated film side facing the latter two samples. The preset distance between the probes is 1.00 mm. Previous experiments have shown that the minimum length for copper azides with a volumetric energy density of 8.45 kJ/cm^3^ (density 2.38 g/cm^3^) to reach a stable detonation state is 1.00 mm [16]. After the first copper azide sample is ignited by the surface-mounted film bridge wire, the detonation wave grows upward and reaches a stable detonation state by the end of the second sample. By measuring the detonation wave propagation process through the 4th and 5th samples, accurate detonation velocity data are ensured to be obtained under stable detonation conditions.

#### 2.5.2. Impact Initiation Test of HNS–IV Explosive

To evaluate the output capability of the prepared copper azide samples and their initiation performance against an insensitive explosive, copper azide impact initiation tests on HNS–IV explosives were conducted. HNS–IV explosive, known for its high thermal stability and low mechanical sensitivity, is often used as a booster explosive in evaluating the output performance of micro-initiation devices [17]. By examining the initiation results of the HNS–IV explosive under different charge thicknesses and flyer conditions using the copper azide micro-charge, combined with the surface trace characteristics and dent depth on the steel witness block, the impact initiation capability of the copper azide micro-charge can be assessed.

**Figure 3:**
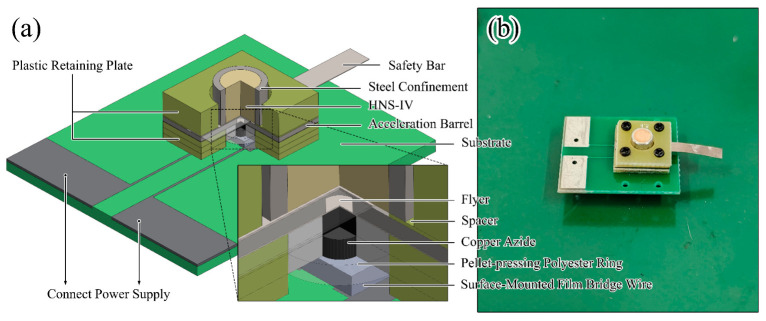
Schematic diagram of the HNS–IV explosive impact initiation test system. (**a**) Schematic; (**b**) Photograph.

A schematic diagram of the HNS–IV explosive impact initiation test system is shown in Figure 3. The system mainly consists of a substrate, a surface-mounted film bridge wire, copper azide samples, plastic retaining plates, a flyer film, a metal acceleration barrel, a spacer, a metal safety bar, an HNS–IV pellet with steel confinement, and a steel witness block. The dimensions of the copper azide sample are the same as those in the detonation velocity test. The plastic retaining plates are 0.50 mm thick, with central hole diameters of 3.00 mm and 4.5 mm. The metal acceleration barrel is 0.40 mm thick, with a central hole diameter of 0.80 mm. The spacer is 0.10 mm thick, designed to reserve space for the movement of the metal safety bar. The metal safety bar is 80 μm thick and made of titanium. The HNS–IV pellet size is Φ3.00 × 3.00 mm, with a density of 1.56 g/cm^3^. Combined with the steel confinement, the overall size is Φ4.00 × 3.00 mm. During the test, the film bridge wire initiates the explosion of the copper azide sample. The detonation wave drives the deformation and movement of the flyer film, causing it to be sheared at the entrance of the acceleration barrel (which has a through-hole) to form a small flyer. This small flyer is further accelerated within the barrel and then impacts the HNS–IV pellet, achieving impact initiation. To ensure safety before placing the device into the explosion chamber, a metal safety bar is inserted between the acceleration barrel end and the HNS–IV pellet to prevent accidental initiation of copper azides from directly detonating the HNS–IV explosive.

## 3. Results

### 3.1. Characterization of Copper Nanoparticles

#### 3.1.1. Regulatory Effect of NTA on Copper Nanoparticles Morphology

**Figure 4:**
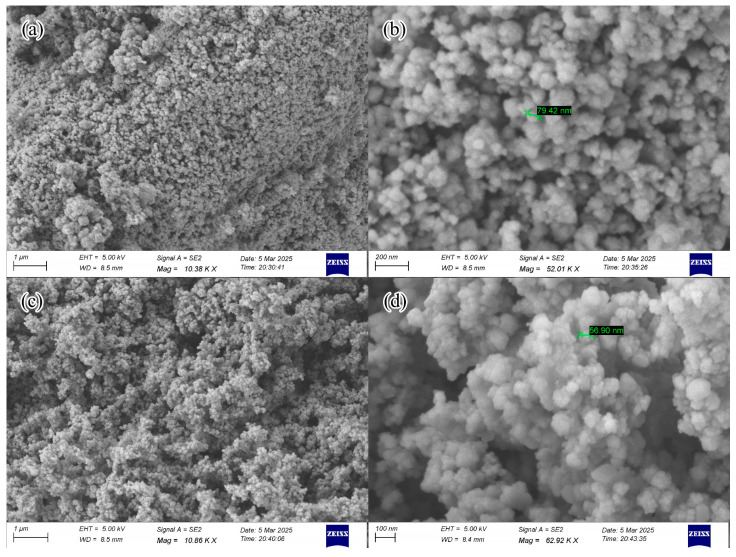
SEM images of copper nanoparticles. (**a**,**b**): Control sample with distilled water; (**c**,**d**): sample prepared with NTA·H·2Na.

To evaluate the regulatory effect of the multidentate complexing agent NTA·H·2Na on the nucleation and growth process of copper nanoparticles, SEM characterization was performed on copper nanoparticle samples prepared with NTA·H·2Na and on control samples where the NTA solution was replaced with an equal amount of distilled water, as shown in Figure 4. SEM results revealed that both groups of samples consisted of agglomerated structures formed by numerous nanoparticles, primarily due to the high surface energy of metallic copper nanoparticles, leading to contact, attraction, and secondary agglomeration during centrifugation, collection, and drying processes. Significant differences were observed between the two groups in terms of primary particle uniformity and agglomeration morphology.

For the sample prepared with NTA·H·2Na (Figure 4c,d), the agglomerates exhibited a relatively loose and porous structural feature overall, with relatively consistent particle scales. High-magnification images revealed numerous quasi-spherical particles, with primary particle sizes qualitatively estimated (from SEM inspection) to be mainly in the 50–100 nm range. Particle boundaries were relatively clear, and phenomena such as neck formation and particle coalescence between particles were weak. This indicates that the nucleation and growth process of copper nanoparticles was subjected to a certain degree of restriction and regulation, resulting in a concentrated particle size distribution.

In contrast, the control sample prepared with an equal amount of distilled water (Figure 4a,b) exhibited a denser blocky agglomerate structure with fewer pores. Low-magnification images showed relatively dense large-scale agglomerated blocks with diameters ranging from 0.5 to 1 μm, suggesting significant fusion and secondary coalescence of particles. High-magnification images revealed, besides primary particles around 50 nm in size, numerous instances of particle fusion and neck formation, locally forming larger secondary particles or agglomerated blocks with sizes ranging from 50 to 200 nm. The particle size distribution broadened significantly, and inter-particle coalescence was more pronounced. This suggests that in the system lacking coordination regulation, the nucleation and growth stages of copper nanoparticles during reduction overlapped strongly, and the newly generated particles had lower stability, making them more prone to collision, coalescence, and subsequent agglomeration.

These morphological differences demonstrate that NTA effectively regulates the nucleation and growth of copper nanoparticles by forming a stable water-soluble Cu(II)-NTA complex precursor with Cu(II). This complexation reduces the effective concentration of free, highly active Cu(II) species in solution, providing a slow-release effect that leads to a more gradual supply of metallic copper atoms. Consequently, the instantaneous supersaturation is reduced and the growth rate is slowed, enabling a more controlled separation of nucleation and growth. Additionally, the coordination adsorption of NTA on the particle surface provides electrostatic and steric stabilization, further reducing coalescence and fusion between particles. In the control system, the absence of these regulatory mechanisms results in a faster, more abrupt reduction with concurrent nucleation and rapid growth, ultimately leading to more pronounced agglomeration and a broader particle size distribution.

#### 3.1.2. Micromorphology and EDS Analysis of Oxidized Samples

**Figure 5:**
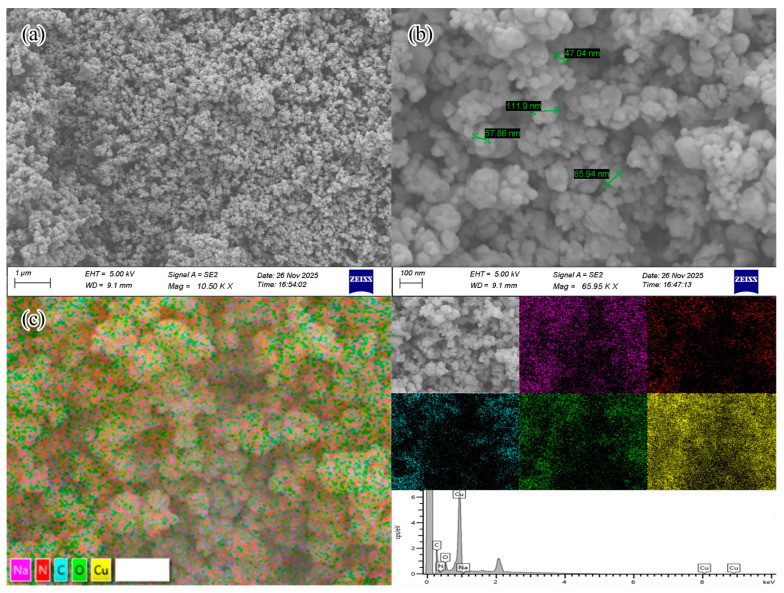
SEM–EDS images of oxidized copper nanoparticle samples. (**a**,**b**) SEM images; (**c**) EDS spectrum.

To investigate the stability of copper nanoparticles in an air environment, copper nanoparticle samples prepared with NTA·H·2Na were placed in air for oxidation over one week. The oxidized samples were then subjected to SEM–EDS analysis, as shown in Figure 5 and Table 3. The SEM images of the oxidized samples still showed nanoparticle agglomerated structures, with small particle sizes primarily on the order of 50 nm. However, compared to samples with a lower degree of oxidation, the particle boundaries appeared slightly blurred, with more instances of 100–150 nm fused and secondary coalesced large particles observed locally, and the overall porosity seemed somewhat reduced. This suggests that some degree of structural reconstruction occurs on the particle surface during air oxidation. The formation of an oxide layer may promote contact and bridging between particles, thereby enhancing the degree of agglomeration.

EDS survey spectrum analysis revealed a clear O signal in the sample besides Cu, indicating surface oxidation of the copper nanoparticles upon air exposure, forming oxygen-containing species. Semi-quantitative results showed approximately 14.54 wt% Cu and 3.30 wt% O, suggesting that oxidation primarily occurred on the particle surface layer and had not yet developed into large-scale bulk oxidation. Na was 0 wt%, indicating minimal alkali metal residues. Simultaneously, strong C and N signals were detected, approximately 44.25 wt% and 37.90 wt%, respectively. On one hand, this may originate from residual NTA ligands and their organic components adsorbed on the particle surface. On the other hand, nanoparticle powder testing typically uses carbon-based conductive tape for fixation, and the excitation volume of the electron beam may include contributions from the substrate, partially contributing to the elevated light element content. As previously demonstrated using the identical synthetic route [15], the as-prepared copper nanoparticles possess a core–shell structure consisting of a metallic Cu core, a Cu_2_O shell, and an outer NTA-derived organic coating. This surface architecture is responsible for the observed oxidation resistance, and the strong C and N signals detected here (Table 3) are consistent with the residual NTA-related species on the particle surface.

#### 3.1.3. DLS Particle Size Analysis of Oxidized Samples

**Figure 6:**
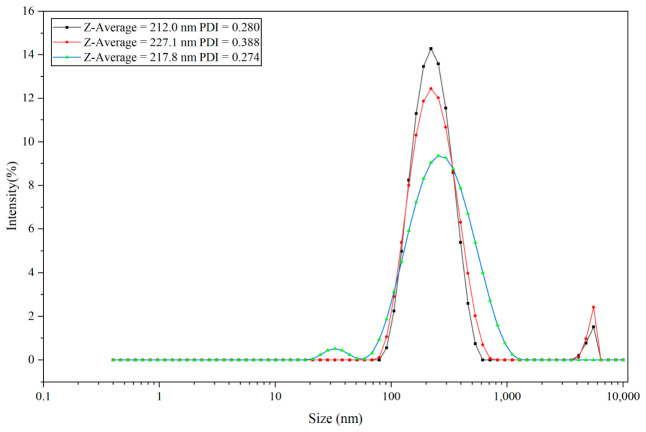
DLS intensity distribution spectra of oxidized samples.

To further assess the particle size characteristics of the oxidized samples, Dynamic Light Scattering (DLS) testing was performed on the air-oxidized copper nanoparticles (after one week of exposure) dispersed in anhydrous ethanol. The suspension was sonicated for 5 min prior to DLS measurement. As shown in Figure 6, the DLS results indicated that the main peak of the oxidized samples shifted towards larger particle sizes overall, with the primary peak located in the range of 237.5–313.0 nm (intensity 96.5–98.0%). The corresponding Z-average results showed a particle size range of 212.0–227.1 nm, with the PDI values of 0.274–0.388, indicating a relatively large and broad hydrodynamic size distribution of the sample in the dispersion system. Among the measurements, a weak secondary peak (intensity 2.5–3.5%) appeared at approximately 5.21–5.30 μm, suggesting the presence of a small amount of large agglomerates in the sample during dispersion. Additionally, in another measurement, a weak secondary peak (intensity 2.0%) appeared at approximately 34.06 nm, possibly related to a small number of primary particles that did not undergo secondary aggregation.

It should be noted that the intensity distribution in DLS is highly sensitive to large particles; a small number of large agglomerates can be amplified in the intensity distribution. Therefore, the micron-scale secondary peaks do not necessarily indicate that these large particles constitute a significant proportion by number. Overall, the oxidized samples predominantly exhibited hydrodynamic sizes on the order of hundreds of nanometers in ethanol, generally larger than the primary particle sizes observed by SEM, yet still showed a certain tendency towards agglomeration under ethanol dispersion conditions. This difference can be attributed to the presence of surface oxide layers, residual organic ligands, and solvation layers on the particles in solution, as well as weak inter-particle aggregation increasing their effective diffusion radius.

From the above results, although both SEM images and DLS measurements indicate that the oxidized samples exhibit some differences in particle size distribution and surface morphology due to surface oxidation, the prepared copper nanoparticles still maintain a relatively uniform particle size distribution and a loose, porous structure after air oxidation. The NTA-derived surface layer provides a certain degree of oxidation resistance. Importantly, the copper nanoparticles used in subsequent gas–solid azidation reactions were freshly prepared samples with minimal air exposure, which maintained high surface energy conducive to their reactivity with HN_3_ gas. The one-week oxidation test primarily demonstrates that the precursor possesses acceptable environmental tolerance under routine handling conditions (weighing, press-loading) without undergoing rapid structural degradation.

### 3.2. Morphological Evolution of Copper Azides

**Figure 7:**
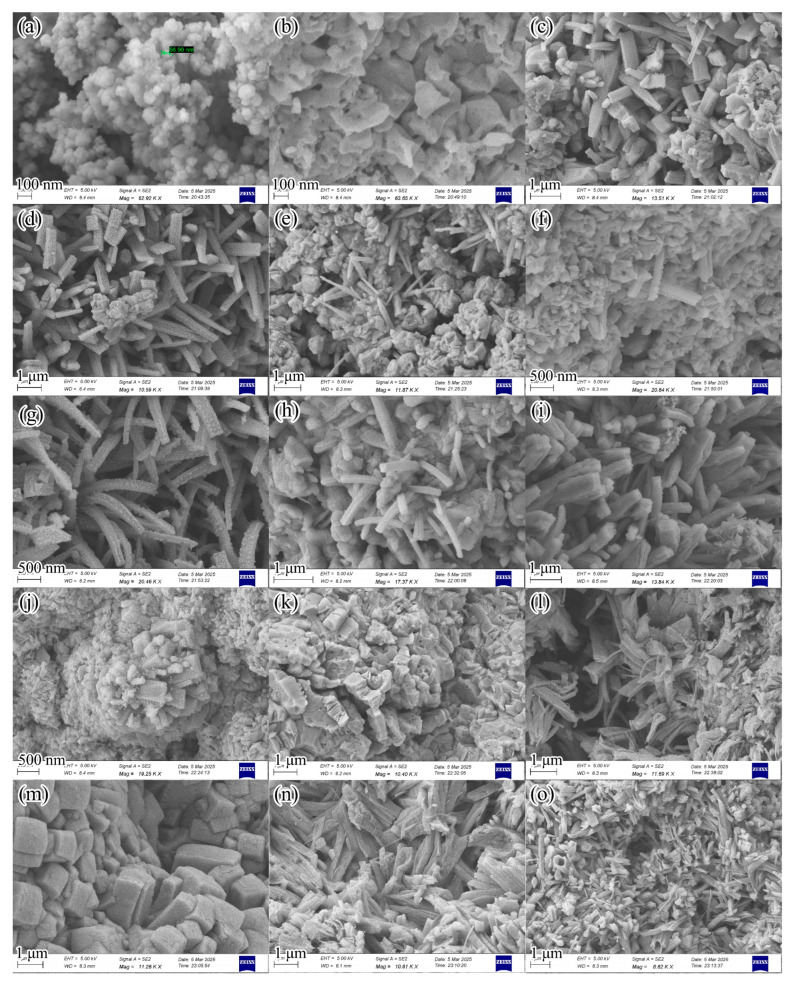
Morphology of copper azide samples at different reaction times. (**a**–**o**) Reaction times: 0 min, 1 min, 2 min, 3 min, 4 min, 5 min, 10 min, 30 min, 1 h, 3 h, 6 h, 12 h, 24 h, 48 h, 72 h.

To evaluate the micromorphological evolution process of copper azides during the gas–solid in situ reaction, SEM characterization was performed on copper azide samples at different reaction times, with results shown in Figure 7. Figure 7a shows the unreacted copper nanoparticles, and Figure 7b–o correspond to reaction times of 1 min, 2 min, 3 min, 4 min, 5 min, 10 min, 30 min, 1 h, 3 h, 6 h, 12 h, 24 h, 48 h, and 72 h, respectively. It should be noted that the observed morphologies from SEM come from limited fields of view on the sample surface, and samples at the same reaction time may be at different evolutionary stages in different regions. Therefore, Figure 7 displays several representative local morphologies. The following description summarizes the overall patterns in terms of “typical morphological units—evolutionary stages”.

Overall, the morphological evolution can be summarized into three stages: early-stage rapid nucleation of product islands and establishment of primary structures (1–5 min), mid-stage anisotropic growth and coalescence (10–60 min), and late-stage continuous product layer formation and structural reconstruction (3–72 h). Notably, two types of typical morphological units coexisted in the early stage of the reaction: one was flat, irregular blocks formed relying on the copper nanoparticle framework; the other was branch-like, dendritic structures with a distinct aspect ratio. These two types of morphologies were interlaced spatially and gradually underwent fusion and coalescence during the subsequent reaction process, ultimately forming contiguous blocky product areas and complex pore structures such as gullies and voids.

(1) Stage I: Product Island Nucleation and Primary Morphology Establishment (1–5 min).

The unreacted copper nanoparticles exhibited a particulate accumulated framework morphology (Figure 7a). At 1 min of reaction (Figure 7b), flat, irregular small blocks relying on the framework could be observed, with the small blocks fusing with each other to form local contiguous areas. Simultaneously, fine particulate protrusions on the order of about 10 nm were visible in the gaps between blocks and on surfaces. These fine protrusions can be regarded as product islands, nuclei, or their coalesced aggregates in the early stage of reaction, indicating that this stage is dominated by high-density nucleation driven by rapid surface reaction. By 2–5 min (Figure 7c–f), besides the continuous coalescence of irregular blocks, micron-scale branch-like structures appeared in some areas, accompanied by features such as gradually deepening granular textures on the branch surfaces and tapering branch tips, suggesting the emergence of pronounced tip-preferred growth and anisotropic growth behavior in localized regions.

(2) Stage II: Branch Tuft Growth and Block–Branch Coalescence Fusion (10–60 min).

At 10–30 min of reaction (Figure 7g,h), branches appeared in tufted distributions and exhibited curvature. The granular protrusions on the branch surfaces continued to grow and underwent fusion connections. Coalescence between branches and surrounding irregular blocks intensified. By 1 h (Figure 7i), branches thickened and fused with neighboring branches. Nanoscale particle clusters or filamentous aggregates were visible at the tips of some branches. Combined with the time-series comparison, these particle clusters and filaments, closely connected to branches and blocks, are more consistent with the morphological characteristics of localized secondary nucleation and secondary growth sites: when reactant supply and diffusion conditions are uneven within micro-regions, crystal defects, steps, or interface positions are more likely to become point-like advancing locations for continuous growth, thereby forming cluster-like protrusions and promoting further branch coalescence.

(3) Stage III: Continuous Blocky Product Formation and Late-Stage Structural Reconstruction (3–72 h).

At 3 h of reaction (Figure 7j), branch coalescence formed contiguous blocky regions with blurred boundaries. At 6–12 h (Figure 7k,l), porous block-like structures and filamentous cluster structures closely attached along the branch growth direction could be observed, indicating that while the product continued to generate and volume expanded, the system might gradually enter a diffusion-controlled state after product layer thickening, the reaction rate decreased, and local flux differences increased, prompting structural rearrangement and retaining growth traces such as pores and gullies. By 24 h (Figure 7m), aggregated regions of relatively regular cubic blocks were visible, suggesting that over longer time scales, the product tends towards a more stable structure through crystal plane preferential exposure and coalescence. At 48–72 h (Figure 7n,o), complex morphologies such as shale-like fusion interfaces, gullies, and hollow tubes appeared in the samples, reflecting further ripening, reconstruction, and pore evolution driven by diffusion limitations and interfacial energy reduction in the late stage.

### 3.3. Detonation Performance of Copper Azides

#### 3.3.1. Detonation Velocity Test Results

**Figure 8:**
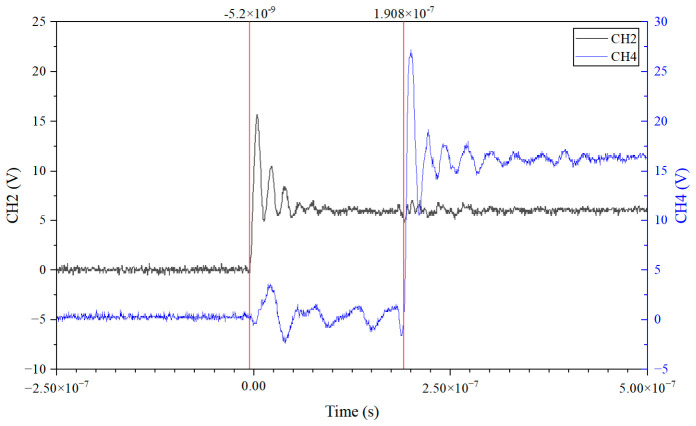
Typical oscilloscope voltage–time curve.

Figure 8 shows the typical oscilloscope voltage–time curve. The figure presents the voltage signal changes in the two conduction circuits, where CH2 records the voltage signal of the circuit that conducts first, and CH4 records the voltage signal of the circuit that conducts later. When the detonation wave propagates to the position of the flexible electrical probe, the high temperature and pressure effects of the detonation products instantly conduct the coated film layer of the electrical probe, causing a pronounced voltage jump across the circuit resistor. The oscilloscope immediately records a rapid rise in the voltage signal. It can be seen from the figure that clear voltage transients appear in the voltage signals of both channels, with their rising edges corresponding to the instant the electrical probe becomes conductive by the detonation products. By reading the time corresponding to the rising edges of the voltage signals from the two channels, the time interval between the conduction of the two probes can be obtained. The oscilloscope measured the time difference as Δ*t* = 196 ns.

The two flexible electrical probes are located behind the 3rd and 5th copper azide samples, respectively. Therefore, the corresponding detonation wave propagation distance is the sum of the thicknesses of the 4th and 5th samples. The thickness of a single copper azide sample measured by a micrometer screw was 0.50 mm. After combining independent random errors, the total thickness of the two samples can be taken as *L* = (1.00 ± 0.014) mm. According to the detonation velocity calculation formula, the average detonation velocity of the copper azide sample under these conditions was calculated to be 5.10 × 10^3^ m/s. Combining the measurement error of the probe spacing and the time resolution of the oscilloscope for error analysis, the uncertainty of this average detonation velocity is approximately 0.07 × 10^3^ m/s. Therefore, the average detonation velocity of the copper azide sample can be expressed as (5.10 ± 0.07) × 10^3^ m/s. This result indicates that, under the present charge structure and test conditions, the copper azides can form a stable detonation and achieve high-velocity propagation.

#### 3.3.2. Impact Initiation Test Results of HNS-IV Explosive

A total of 4 groups of HNS-IV explosive impact initiation tests were conducted. In all test groups, the acceleration barrel length was 0.40 mm, and the air gap length was 0.10 mm. Only the number of copper azide samples and the flyer material were varied. The thickness of a single copper azide sample was 0.50 mm, so the total thickness for two stacked samples was 1.00 mm. The test results are shown in Table 4. When using two copper azide samples to drive a 30 μm Ti flyer, and when using a single copper azide sample to drive a 30 μm PI flyer, the HNS-IV explosive was not successfully initiated. In contrast, when using two copper azide samples to drive a 30 μm PI flyer, and when using two copper azide samples to drive a 40 μm Ti flyer, the HNS-IV explosive was successfully initiated.

**Figure 9:**
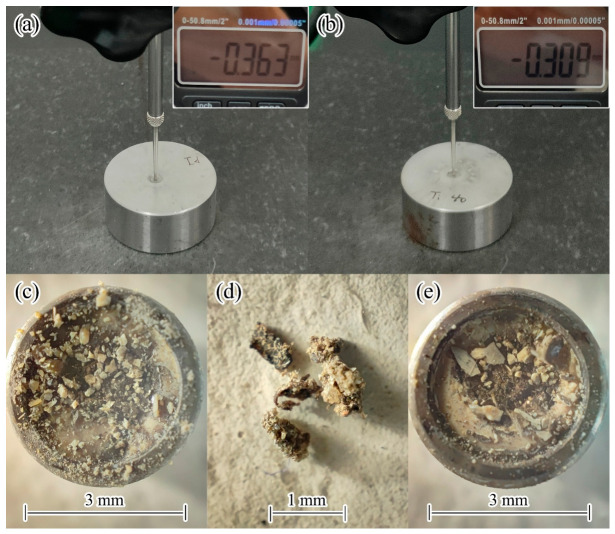
Results of the HNS-IV explosive impact initiation test. (**a**) Steel witness block after successful initiation with a 30 μm PI flyer; (**b**) steel witness block after successful initiation with a 40 μm Ti flyer; (**c**) HNS-IV pellet that failed to detonate under a 30 μm Ti flyer; (**d**) recovered fragmented 30 μm Ti flyer; (**e**) HNS-IV pellet that failed to detonate under a 30 μm PI flyer.

Photographs and measurement results of the steel witness blocks after successful initiation tests are shown in Figure 9a,b. After initiation, a pronounced dent formed on the surface of the steel witness block, indicating that the HNS-IV explosive underwent effective detonation upon flyer impact. Photographs of the HNS-IV pellet and recovered fragmented 30 μm Ti flyer after failed initiation tests are shown in Figure 9c–e. From the morphology of the failed samples, it can be seen that when initiation was unsuccessful, the HNS-IV pellet exhibited superficial impact damage and localized ablation traces on the pellet surface, without forming a stable detonation. The fragmentation of the recovered 30 μm Ti flyer indicates that flyer integrity is an important factor affecting initiation reliability.

These results demonstrate that the impact initiation capability of the copper azide samples is closely related to charge thickness and flyer parameters. Increasing the charge thickness from 0.50 mm to 1.00 mm enabled successful initiation with the 30 μm PI flyer, consistent with the detonation velocity results showing stable detonation at 1.00 mm charge length. For the Ti flyer under 1.00 mm charge conditions, the 30 μm flyer failed whereas the 40 μm flyer succeeded, suggesting that flyer material and thickness significantly affect initiation outcomes through their influence on structural integrity during shearing and acceleration.

## 4. Discussion

### 4.1. Morphological Comparison of Copper Azides with Previous Studies

The three-stage morphological evolution observed in this study—product island nucleation, branch/block coalescence, and continuous product layer formation—is generally consistent with the gas–solid azidation framework of “product island formation—continuous product layer formation—Ostwald ripening” established by Ren et al. [8]. However, a notable difference lies in the pronounced dendritic growth observed in this study, in contrast to the predominantly blocky coalescence reported in Ref. [8] under similar low-temperature, high HN_3_ concentration conditions. Despite this morphological variation, the identical reaction conditions (60 °C, high HN_3_ partial pressure) and precursor type suggest that the two-step azidation pathway remains unchanged, and the final product after 24–72 h is therefore predominantly CuN_3_, consistent with the phase composition reported in Ref. [8]. This morphological divergence likely arises from the microstructural characteristics of the copper nanoparticle packing system.

The particle size distribution (50–100 nm), neck formation, and secondary coalescence of the copper nanoparticles used in this work create a packing bed with highly non-uniform curvature, defect density, and inter-particle connectivity. These microstructural features lead to spatial differentiation in local nucleation density: regions with higher curvature and more exposed active sites favor high-density nucleation and block growth, whereas regions where pore diffusion within the packing bed establishes HN_3_ concentration gradients experience reduced effective HN_3_ partial pressure, thereby lowering nucleation density and allowing a few nuclei to develop into high-aspect-ratio branch structures. Additionally, the surface oxide layer (Cu_2_O, CuO) and residual organic species on the copper nanoparticle samples may further modulate local reactivity by shielding exposed metallic copper active sites and altering gas diffusion paths, contributing to the coexistence of block and branch growth modes.

This dual-mode growth mechanism is consistent with findings by Wang et al. [6,18], who reported a similar “product island—branch elongation—lateral coalescence” pattern for nanoporous copper precursors with particle sizes of 50–60 nm and existing neck connections. The agreement across studies suggests that precursor particle scale and inter-particle connectivity are important factors influencing the morphological evolution of copper azides during in situ gas–solid synthesis.

### 4.2. Evaluation of Detonation Performance

The average detonation velocity measured in this work, (5.10 ± 0.07) × 10^3^ m/s, is in good agreement with the stable detonation limit velocity of 5164 m/s reported by Li et al. [16] for copper azide micro-charges. The small discrepancy (approximately 1.2%) can be attributed to variations in charge density (1.50 g/cm^3^ in this work) and confinement conditions between the two studies. This agreement validates the in situ synthesis route established here as capable of producing copper azide micro-charges with detonation performance comparable to those prepared by other methods. Furthermore, the achievement of stable detonation at a charge thickness of 1.00 mm confirms that the copper azides prepared via this aqueous-phase precursor route possess a sufficiently short detonation growth distance for micro-initiation applications.

### 4.3. Factors Affecting Impact Initiation Reliability

The impact initiation results indicate that the initiation capability of the copper azide samples towards the HNS-IV explosive is closely related to charge thickness and flyer parameters. When the total charge thickness was 1.00 mm, both the 30 μm PI flyer and the 40 μm Ti flyer successfully initiated the HNS-IV explosive, whereas initiation failed with a 0.50 mm charge driving a 30 μm PI flyer. For the Ti flyer under 1.00 mm charge conditions, the 30 μm flyer did not achieve successful initiation in this experiment.

It should be noted that the 30 μm Ti flyer does not lack the ability to initiate the HNS-IV explosive per se. The existing literature [18] reports that under conditions with a copper azides charge size of Φ1.0 mm × 1.0 mm and an acceleration barrel length of 0.50 mm, a 30 μm Ti flyer can successfully initiate the HNS-IV explosive, indicating that this flyer configuration possesses a certain initiation capability. Concurrently, relevant experiments by our research group show that under conditions with an acceleration barrel length of 0.20 mm and an air gap length of 0.25 mm, the success rate for two copper azide samples driving a 30 μm Ti flyer to initiate the HNS-IV explosive was only 70%, suggesting that the initiation outcome under these conditions exhibits some discreteness and relatively insufficient reliability.

The fragmentation of the 30 μm Ti flyer recovered after the failed test in this experiment, combined with the results from Ref. [16] where the PDV velocity measurement signal for a 30 μm Ti flyer driven by two copper azide samples was poor, with extensive signal dispersion appearing after the flyer velocity stabilized, suggests that the 30 μm Ti flyer is prone to fragmentation or integrity degradation during the explosion-driven shearing and acceleration process. This would result in dispersed impact loading upon striking the HNS-IV explosive, thereby affecting the stability of the initiation outcome. Therefore, the 30 μm Ti flyer does not lack the ability to initiate the HNS-IV explosive per se, but its initiation reliability is insufficient under the structural conditions investigated in this study.

When the Ti flyer thickness was increased to 40 μm, its structural integrity and resistance to fragmentation improved, allowing it to impact the HNS-IV pellet in a more intact form and achieve initiation. In contrast, the 30 μm PI flyer successfully initiated the HNS-IV explosive under the same total charge thickness conditions, indicating that flyer material significantly influences shearing formation characteristics, flight stability, and impact energy transfer efficiency. These results collectively demonstrate that properly matching the output capability of the copper azide micro-charge with the flyer structural parameters is key to achieving stable impact initiation of the HNS-IV explosive.

## 5. Conclusions

This paper systematically investigated the preparation of copper nanoparticles, the construction of precursors for gas–solid in situ azidation reaction, the in situ synthesis of copper azides, and the characterization of their morphology and detonation performance. The main conclusions are as follows:

(1) Copper nanoparticles were successfully prepared at room temperature under air atmosphere using an NTA·H·2Na coordination-assisted aqueous reduction method. SEM characterization results showed that, compared with control samples prepared without NTA, the primary particles of copper nanoparticles obtained with NTA·H·2Na exhibited more uniform size, clearer boundaries, significantly weakened neck formation and fusion phenomena between particles, and the overall agglomerates presented a relatively loose and porous structural feature. This indicates that NTA, by forming a complex precursor with Cu(II), effectively regulates the nucleation and growth process of copper nanoparticles during reduction, which is beneficial for inhibiting rapid particle growth and secondary coalescence.
(2) SEM, EDS, and DLS characterization results of oxidized samples after one week of air exposure showed that copper nanoparticles undergo a certain degree of surface oxidation in air, but the oxidation is mainly confined to the particle surface layer and has not developed into large-scale bulk oxidation. The oxidized samples still maintained a nanoparticle agglomerated structure in morphology, though particle boundaries were slightly blurred, and local fusion and secondary agglomeration were somewhat enhanced. Comprehensive analysis indicates that the prepared copper nanoparticles possess acceptable structural stability under routine operating conditions and can meet the requirements for use as precursors in subsequent reactions.
(3) An experimental apparatus and method were established for the in situ synthesis of copper azides by generating HN_3_ gas from the reaction of NaN_3_ with concentrated phosphoric acid, followed by a gas–solid reaction with copper nanoparticles. SEM characterization of samples at different reaction times indicated that the formation process of copper azides could be summarized into three stages: early-stage rapid nucleation of product islands and establishment of primary structures, mid-stage coalescence and growth of branch and block structures, and late-stage structural reconstruction after the formation of a continuous product layer. Two typical morphological units—flat irregular blocks and branch-like structures—were simultaneously observed during the reaction. Their coexistence and evolution reflect the significant influence of spatial non-uniformity in local nucleation density, material transport conditions, and interfacial states within the particle packing system on the product morphology.
(4) The detonation velocity of the copper azide samples was tested using the electrical probe method. The results showed that under the charge structure and test conditions established in this paper, the average detonation velocity of the copper azide samples was (5.10 ± 0.07) × 10^3^ m/s, which is close to the value reported in the literature, indicating that the prepared samples can form a stable detonation and achieve high-velocity propagation.
(5) Flyer impact initiation tests demonstrated that the copper azide samples could effectively initiate the HNS-IV explosive when the total charge thickness reached 1.00 mm with appropriate flyer parameters. Both 30 μm PI and 40 μm Ti flyers achieved successful initiation, whereas 0.50 mm charge thickness or 30 μm Ti flyer resulted in initiation failure due to insufficient output energy or degraded flyer integrity. These results indicate that properly matching the charge output capability with flyer structural parameters is essential for reliable impact initiation of the HNS-IV explosive.

## Figures and Tables

**Table 1:** Main chemical reagents.

Reagent	Purity	Manufacturer	Address
NTA·H·2Na	98%	Shanghai Aladdin Biochemical Technology Co., Ltd.	Shanghai, China
Cu(OH)_2_	95%	Shanghai Macklin Biochemical Technology Co., Ltd.	Shanghai, China
N_2_H_4_·H_2_O	AR (80 wt%)	Sinopharm Chemical Reagent Co., Ltd.	Shanghai, China
NaN_3_	AR	MYM Biological Technology Co., Ltd.	Osaka, Japan
H_3_PO_4_	AR (85 wt%)	Shanghai Saan Chemical Technology Co., Ltd.	Shanghai, China
NaOH	AR	Shanghai Aladdin Biochemical Technology Co., Ltd.	Shanghai, China
CaCl_2_	AR	ZanCheng (Tianjin) Technology Co., Ltd.	Tianjin, China
C_2_H_5_OH	AR	Shanghai Macklin Biochemical Technology Co., Ltd.	Shanghai, China
N_2_	AR	Beijing Huanyu Jinghui Gas Technology Co., Ltd.	Beijing, China

**Table 2:** Main experimental instruments.

Instrument	Model	Manufacturer	Address
Magnetic Stirrer	ZNCL–GS130 × 60	Tianjin Yongda Chemical Reagent Co., Ltd.	Tianjin, China
High–Speed Centrifuge	H1850	Shanghai Bangxi Instrument Technology Co., Ltd.	Shanghai, China
Ultrasonic Cleaner	KQ–300E	Shanghai Yiheng Scientific Instrument Co., Ltd.	Shanghai, China
Vacuum Drying Oven	DZF–6020	Shanghai Kesheng Instrument Co., Ltd.	Shanghai, China
Automatic Tablet Press	PP–20S	Tianjin Nuolaixinda Technology Co., Ltd.	Tianjin, China
Electronic Balance	AD 6 Autobalance	PerkinElmer, Inc.	Waltham, MA, USA
Rotary Evaporator	IKA RV 10 digital	IKA–Werke GmbH & Co. KG	Staufen im Breisgau, Germany
Field Emission Scanning Electron Microscope (with EDS)	GeminiSEM 360	Carl Zeiss AG	Oberkochen, Germany
Nanoparticle Size and Zeta Potential Analyzer	Malvern ZetasizerNano ZS90	Malvern Panalytical Limited	Malvern, UK
Surface-Mounted Film Bridge Wire	JMC0805–2R0	Nanjing Jingchu Electronic Technology Co., Ltd.	Nanjing, China
DC Power Supply	P3005D	Shenzhen Leda Precision Tools Co., Ltd.	Shenzhen, China
Digital Oscilloscope	DPO3034	Tektronix, Inc.	Beaverton, OR, USA

**Table 3:** Elemental content distribution from the overall spectrum.

Element	Signal Type	Wt%	Wt% Sigma
C	EDS	44.25	0.05
N	EDS	37.90	0.05
O	EDS	3.30	0.03
Na ^1^	EDS	0.00	0.03
Cu	EDS	14.54	0.08
Total		100.00	

^1^ The 0.00 wt% value for Na indicates that the sodium content was below the detection limit of the EDS measurement, not a true zero concentration.

**Table 4:** Results of copper azide impact initiation of HNS-IV explosive under different conditions.

Total Charge Thickness/mm	Flyer Material	Flyer Thickness/μm	Initiation Result
1.00	Ti	30	No
0.50	PI	30	No
1.00	PI	30	Yes
1.00	Ti	40	Yes

## Data Availability

The data presented in this study are available on request from the corresponding author.
